# 50th Anniversary of the Graduate Program in Nursing, University of São Paulo School of Nursing

**DOI:** 10.1590/1980-220X-REEUSP-2023-0370en

**Published:** 2023-12-22

**Authors:** Ana Luíza Vilela Borges, Maria de La Ó Ramallo Veríssimo, Lucia Yasuko Izumi Nichiata

**Affiliations:** 1 Universidade de São Paulo, Escola de Enfermagem, Departamento de Enfermagem em Saúde Coletiva, São Paulo, SP, Brazil.; 2 Universidade de São Paulo, Escola de Enfermagem, Departamento de Enfermagem Materno-Infantil e Psiquiátrica, São Paulo, SP, Brazil.

The Graduate Program in Nursing (PPGE), of the University of São Paulo School of Nursing (EEUSP), celebrates its 50^th^ anniversary in 2023!

The creation of PPGE is the beginning of the *strict sensu* Graduate Program of EEUSP, as well as the beginning of post-graduation level in the Nursing field in Brazil^(1,2).^

In November 1972, the first meeting of the EEUSP’s Graduate Commission was held, with the pioneering Professors Wanda de Aguiar Horta, Leda Ulson Mattos, and Glete de Alcântara, the latter elected President of the Commission. The master’s course began in the following year. The first dissertation was defended at the PPGE-EEUSP in 1975, with the title “Influence of the level of training of the nursing staff and the checking time of manual counting of arterial pulse frequencies,” authored by Magali Roseira Boemer, later Professor at the University of São Paulo Ribeirão Preto School of Nursing.

PPGE-EEUSP doctoral program began only in 1989 as one of the first courses PhD programs in Brazil. The first thesis was defended in 1993, with the title “Construção e Validação de um programa sobre comunicação não verbal para enfermeiros,” authored by Maria Júlia Paes da Silva, later Professor at EEUSP. In the same year, the first foreign candidate (Chile) was Luz Angélica Muñoz Gonzalez, with the thesis “A doença veio para ficar,” an ethnographic study of the experience of people with diabetes. Doctoral graduates from PPGE have created many other graduate programs in Brazil ever since, which denotes one of its strengths, that is the placement of its alumni in several public and private universities across Brazil. Furthermore, EEUSP supervisors conceived four other graduate programs at this institution.

Since then, more than 1000 masters and 350 doctors have been trained in the Program! These graduates founded other Graduate Programs in Nursing, including at USP itself, in various parts of Brazil, and in other Latin American countries.

Currently, the program consists of two Areas of Knowledge: 1) Health Care, bringing together the Research Fields “Theoretical and Methodological Foundations of Child and Family Health,” “Models and Practices of Care in Women’s, Maternal and Neonatal Health,” and “Policies and Practices in Mental Health”; and 2) Collective Health Nursing, with the Research Fields “Public Health Policies and Workforce Training in Collective Health Nursing” and “Theories and Practices of Collective Health Nursing”.

Along these 50 years, researchers training and studies’ results have contributed to the consolidation of areas of knowledge in Nursing and to the achievement of better levels of health for individuals, families and communities. They have also collaborated with the consolidation of the Brazilian Unified Health System with such academic and technical impact. Corroborating this statement, we recall that, since the beginning of CAPES evaluation, the PPGE-EEUSP had achieved grade 5 and, recently, grade 6, attributed only to programs of academic excellence.

As PPGE-EEUSP turns half a century and looking forward to the future, old and new challenges are posed. Researchers and supervisors must respond to new emergencies, including climate issues and displacement of population groups, without old problems having been resolved, such as the social inequalities and the persistence of neglected diseases. Another challenge is to generate a social impact capable of influencing the health status at the local level, but which is also capable of proposing solutions to be implemented at the global level, considering that health problems are increasingly transversal and common to different populations and regions. It takes agility to recognize social problems, propose solutions, test them, and make them scale-up in increasingly short times, with bold and creative research questions. It is also necessary to develop and validate new theories of Nursing that meet health needs in Brazil and can dialogue with other countries.

With its experienced Faculty, but also in permanent renewal, PPGE-EEUSP reconciles its tradition with the new challenges imposed by the increasingly precarious ways of living and working, and seeks to use the new digital and intelligence tools to improve general health in the coming decades.

We renew our hope that we will continue to strengthen the cornerstones of the PPGE-EEUSP, the unconditional link with the production of Nursing Science, in order to boost the advancement of innovative knowledge and generate transformations in health care and management policies and practices in Nursing and Health.

We invite health workers, researchers and nurses to learn more about the Program^(3).^

Greeting to everyone who made and still make the history of PPGE-EEUSP! “Viva!”.
